# Enhanced Photocatalytic Performance under Visible and Near-Infrared Irradiation of Cu_1.8_Se/Cu_3_Se_2_ Composite via a Phase Junction

**DOI:** 10.3390/nano7010019

**Published:** 2017-01-18

**Authors:** Li-Na Qiao, Huan-Chun Wang, Yang Shen, Yuan-Hua Lin, Ce-Wen Nan

**Affiliations:** State Key Laboratory of New Ceramics and Fine Processing, School of Materials Science and Engineering, Tsinghua University, Beijing 100084, China; qln13@mails.tsinghua.edu.cn (L.-N.Q.); wanghc12@mails.tsinghua.edu.cn (H.-C.W.); shyang_mse@mail.tsinghua.edu.cn (Y.S.); cwnan@mail.tsinghua.edu.cn (C.-W.N.)

**Keywords:** Cu_1.8_Se/Cu_3_Se_2_ composite, visible, near-infrared, photocatalysis, phase junction

## Abstract

A novel Cu_1.8_Se/Cu_3_Se_2_ composite photocatalyst was prepared by the simple precipitation method. This composite possesses a wide photoabsorption until the range of near-infrared light, and exhibits significantly enhanced photocatalytic activity for methyl orange degradation under visible and near-infrared light irradiation compared with bare Cu_1.8_Se and Cu_3_Se_2_. The mechanism of this outstanding photocatalytic behavior can be explained by the calculated energy band positions. The efficient charge separation via a phase junction of Cu_1.8_Se/Cu_3_Se_2_ composite would make a great contribution to its much-enhanced photocatalytic efficiency.

## 1. Introduction

Semiconductor photocatalysis for the degradation of organic pollutants using solar light energy is a promising approach to solving environmental issue. Since TiO_2_ was first reported on photocatalytic water splitting under ultraviolet (UV) light [1], photocatalysts have been widely studied in recent years [2,3,4,5]. Recently, photocatalysts with outstanding photocatalytic properties have been found, such as TiO_2_ and ZnO in the UV spectral range [6,7], and CdS and Bi_2_WO_6_ in the visible range [8,9], and only few semiconductors present near-infrared (NIR) photoactivity, such as Cu_2_(OH)PO_4_ and WS_2_ [10,11]. Nowadays, the development of effective ways to exploit near-infrared light (which accounts for more than 50% of solar energy) and develop UV-visible-near-infrared (UV-Vis-NIR) broad light spectrum photocatalysts is an important emerging topic [12]. Forming heterogeneous structures with matched band potentials is one approach [13,14,15]. For example, Au-tipped PbSe/CdSe/CdS core/shell/shell heterostructure nanocrystals are active NIR photocatalysts for the degradation of methylene blue (MB) [16], and Bi_2_WO_6_/TiO_2_ heterojunction can harness UV, visible, and near-infrared light, and exhibits enhanced broad-spectrum photocatalytic properties to decompose methyl orange (MO) [17]. Thus, the design of a broad light spectrum photocatalyst with heterogeneous structure is of great significance.

Selenides of copper are metal chalcogenide semiconductors with different compositions (Cu_2_Se, Cu_3_Se_2_, CuSe, and Cu_1.8_Se) and various crystallographic forms, which are usually dependent on the preparation process [18,19,20,21,22,23,24,25]. The thermal stability and band gaps of copper selenides vary with their compositions or phases [19,20,21]. These semiconductors with p-type conductivity and direct band gaps have wide applications in solar cells, superconductors, thermoelectric, and photoelectric transformers [26,27,28,29,30]. Therefore, considerable progress on the study of copper selenides has been made, and a number of methods for the synthesis of copper selenides have been explored, such as microwave-assisted heating, sonochemical method, hydrothermal method, and vacuum evaporation [31,32,33,34].

Herein, we report on the aqueous precipitation synthesis of Cu_1.8_Se/Cu_3_Se_2_ heterogeneous structure, bare Cu_1.8_Se, and Cu_3_Se_2_ based on the redox reaction between alkaline selenium solution and cupric aqueous solution under atmospheric pressure. Compared with other reported methods, this method established in aqueous solution is rather simple and safe. The photocatalytic degradation of MO under visible and near-infrared (NIR) light demonstrated that Cu_1.8_Se/Cu_3_Se_2_ composite exhibits much enhanced photocatalytic activity compared with bare Cu_1.8_Se or Cu_3_Se_2_. The mechanism of enhanced photocatalytic activity for Cu_1.8_Se/Cu_3_Se_2_ composite based on the calculated energy band positions was also proposed.

## 2. Results and Discussion

**Figure 1:**
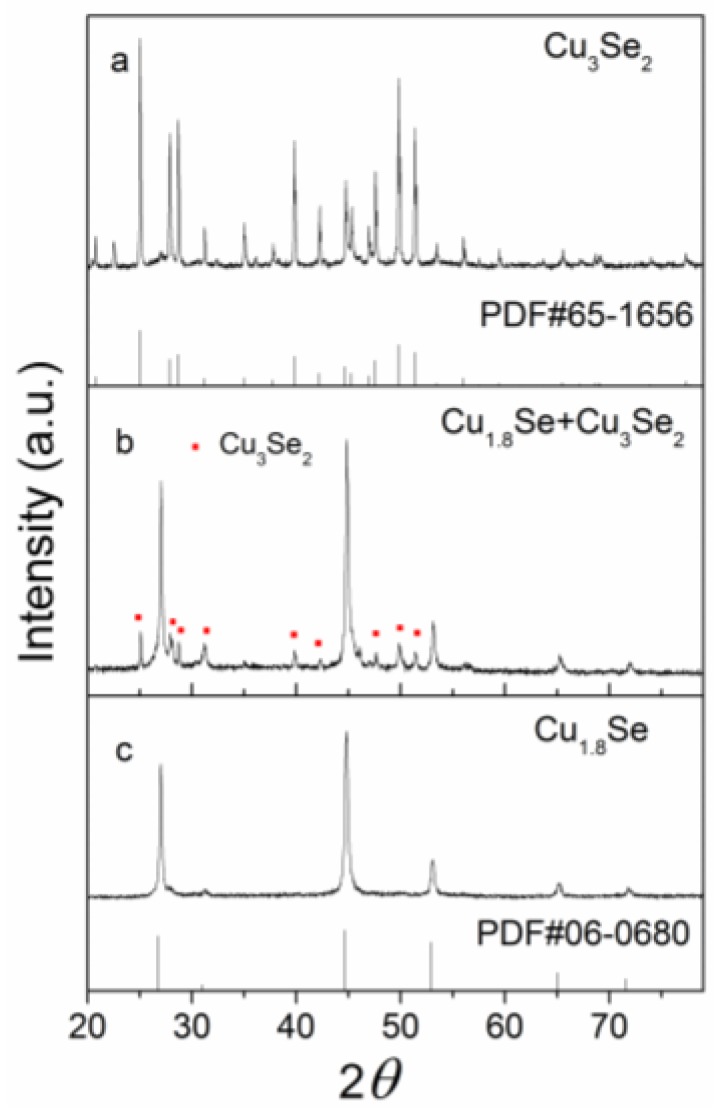
X-ray diffraction profiles of as-prepared copper selenides, (**a**) Cu_3_Se_2_, (**b**) Cu_1.8_Se/Cu_3_Se_2_ composite, (**c**) Cu_1.8_Se.

The composition of products was controlled by varying the addition of NaOH. As shown in Figure 1, when 0.4 g NaOH was added, the X-ray diffraction (XRD) pattern of Figure 1a matched the JCPDS reference file for tetragonal Cu_3_Se_2_ [PDF#65-1656]. When 4 g NaOH was included, the diffraction profile of Figure 1b shows that the composite was composed of tetragonal Cu_3_Se_2_ [PDF#65-1656] and cubic Cu_1.8_Se [JCPDF 06-0680]. By increasing the addition of NaOH to 8 g, the XRD pattern of Figure 1c indicates that all the peaks can be well indexed to cubic Cu_1.8_Se [JCPDF 06-0680]. No characteristic peaks of any impurities were detected in the patterns, and the high diffraction intensity reveals that all the samples had good crystallinity. In addition, the particle sizes calculated from Scherrer’s equation and the BET (Brunauer-Emmett-Teller) specific surface area of different samples are shown in Table 1. There are notbig difference in their particle sizes, and Cu_1.8_Se displays a larger specific surface area than Cu_1.8_Se/Cu_3_Se_2_ composite and Cu_3_Se_2_, which could lead to the exposure of more active sites for the photocatalytic experiment.

**Figure 2:**
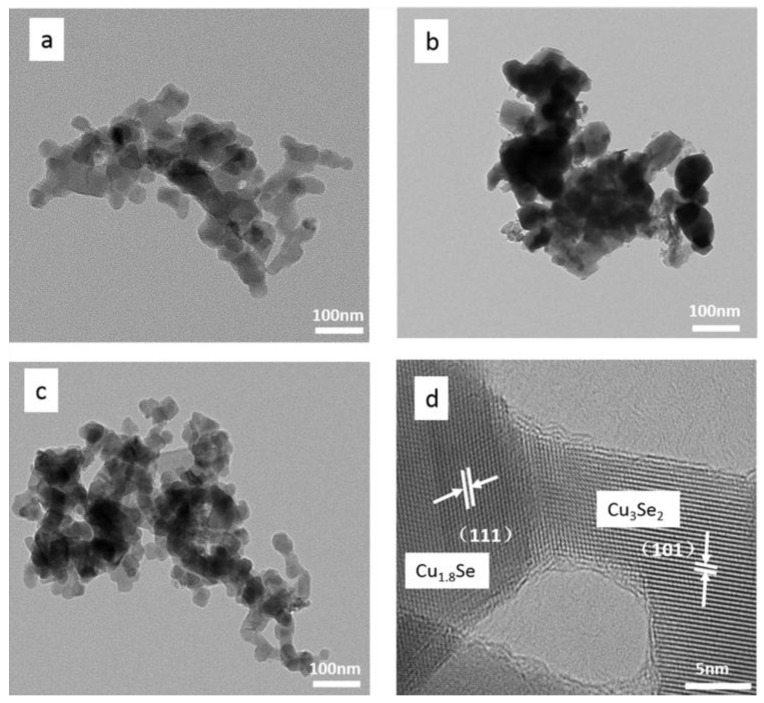
TEM (transmission electron microscopy) images of (**a**) Cu_1.8_Se; (**b**) Cu_3_Se_2_; (**c**) Cu_1.8_Se/Cu_3_Se_2_ composite; (**d**) HRTEM (high resolution transmission electron microscopy) of Cu_1.8_Se/Cu_3_Se_2_ composite.

The morphology and microstructure of Cu_1.8_Se/Cu_3_Se_2_ composite are shown in the TEM (transmission electron microscopy) image of Figure 2c, with homogeneous size distribution of about 50–80 nm (in agreement with the calculated particle size in Table 1), and the bare Cu_1.8_Se (Figure 2a) and Cu_3_Se_2_ (Figure 2b) nanocrystals (NCs) have similar morphologies. The lattice resolved HRTEM (high resolution transmission electron microscopy) image of Cu_1.8_Se/Cu_3_Se_2_ composite indicates the longitudinal fringe spacing of 0.331 nm and the horizontal fringe spacing of 0.354 nm (as seen in Figure 2d), which is consistent with the spacing of (111) and (101) planes of cubic Cu_1.8_Se and tetragonal Cu_3_Se_2_, respectively. The high-quality image of the interface between Cu_1.8_Se and Cu_3_Se_2_ can be observed clearly, which could be considered as a phase junction structure [35].

**Figure 3:**
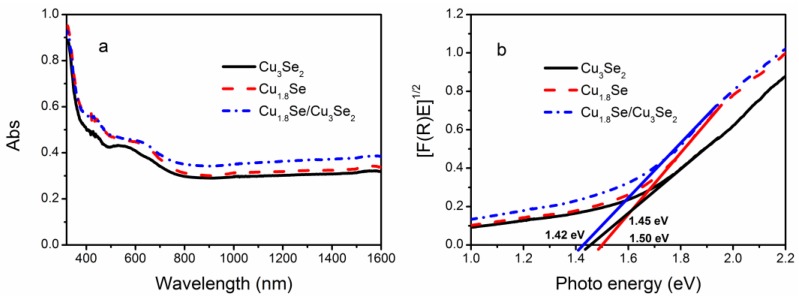
(**a**) UV-visible-near-infrared (UV-Vis-NIR) diffuse reflectance absorption (DRS) spectrum of copper selenides; (**b**) A plot transformed according to the Kubelka–Munk function versus energy of light.

The UV-Vis diffuse reflection spectrum (DRS) of the as-prepared samples is shown in Figure 3a. It can be seen that these samples present effective optical absorption from the UV light region to the whole range of NIR light, which can be attributed to their small bandgaps. As shown in Figure 3b (converted from the DRS spectrum according to the Kubelka–Munk function [36,37,38]), the bandgap energies (*E*_g_) of Cu_1.8_Se/Cu_3_Se_2_ composite, Cu_3_Se_2_, and Cu_1.8_Se were estimated to be around 1.42, 1.45, and 1.5 eV, respectively, which are similar to the reported bandgap [31,32]. It is worth noting that these bandgap values are very close, and Cu_1.8_Se/Cu_3_Se_2_ composite has a relatively narrower band gap and stronger light absorption than that of Cu_3_Se_2_ and Cu_1.8_Se.

**Figure 4:**
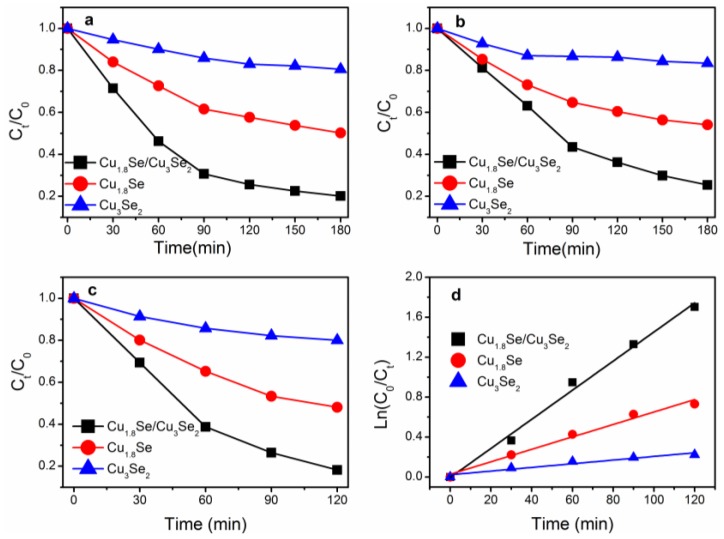
Photocatalytic degradation of methyl orange (MO) solution with copper selenides as catalysts under (**a**) visible light; (**b**) NIR light; (**c**) full solar light; (**d**) the degradation kinetics by means of plotting ln(*C*_0_/*C_t_*) versus time.

**Figure 5:**
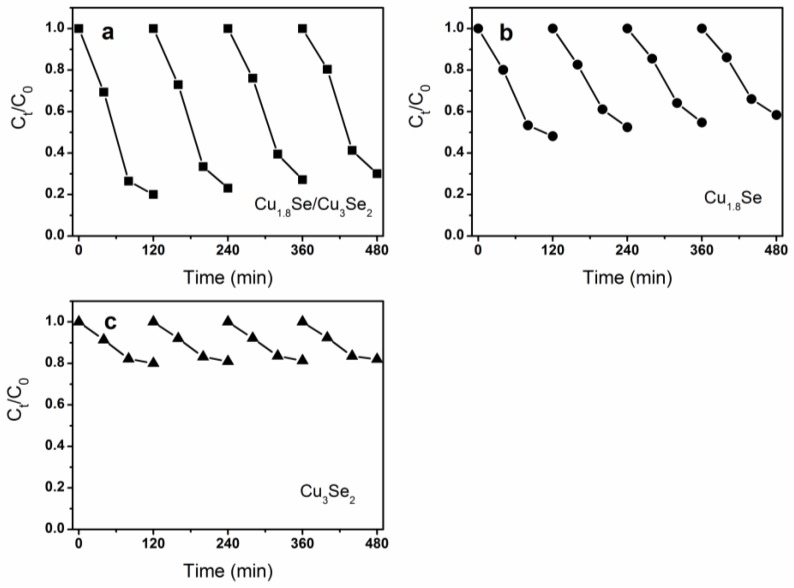
Cycling runs for the photocatalytic degradation of MO under full solar light in the presence of (**a**) Cu_1.8_Se/Cu_3_Se_2_ composite; (**b**) Cu_1.8_Se; (**c**) Cu_3_Se.

The photocatalytic activities of these samples were evaluated by the photodegradation of MO under visible and NIR light irradiation. As shown in Figure 4a,b, under identical experimental conditions, the Cu_1.8_Se/Cu_3_Se_2_ composite exhibited strongly enhanced photocatalytic activity compared to both bare Cu_1.8_Se and Cu_3_Se_2_. The photodegradation rate of MO reached 80% and 75% in the presence of Cu_1.8_Se/Cu_3_Se_2_ composite after 3 h of visible and NIR light irradiation, respectively. Thus, the Cu_1.8_Se/Cu_3_Se_2_ composite shows relatively good visible-NIR broad light spectrum photocatalytic property, which is comparative with that of WS_2_ [11] or Bi_2_WO_6_/TiO_2_ [17]. By contrast, 50% and 46% of MO was degraded by bare Cu_1.8_Se, whereas only 20% and 17% of MO was degraded by bare Cu_3_Se_2_ within the same time. Moreover, photodegradation of MO under full solar light without optical filter was performed. As shown in Figure 4c, after 2 h of full solar light irradiation, 82%, 52%, and 20% of the MO was degraded with Cu_1.8_Se/Cu_3_Se_2_ composite, pure Cu_1.8_Se, and Cu_3_Se_2_ as catalysts, respectively. In addition, the stabilities of these photocatalysts are shown in Figure 5. After four successive cycles, the photodegradation rate of MO under full solar light slightly decreased to 70%, 42%, and 18% within 120 min for Cu_1.8_Se/Cu_3_Se_2_ composite, Cu_1.8_Se, and Cu_3_Se_2_, respectively, indicating that these materials could be re-used without appreciable loss of photocatalytic ability.

To further understand the reaction kinetics of MO degradation under full solar light, we applied the Langmuir−Hinshelwood model, which is well-designed for photocatalytic experiments when the concentration of the organic pollutant is in the millimolar range [39], as expressed by

ln(*C_0_*/*C_t_*) = *kt* (1)

where *C*_0_ and *C_t_* are the concentrations of pollutant in solution at time *t*_0_ and *t*, respectively, and *k* is the kinetic constant, which is calculated to be 0.0145, 0.0062, and 0.0018 min^−1^ for Cu_1.8_Se/Cu_3_Se_2_ composite, bare Cu_1.8_Se, and Cu_3_Se_2_, respectively (Figure 4d). Then, the apparent reaction rate constant (*K*) was calculated to get deeper insight into the photodegradation rate per unit surface area according to the following formula:

*K* = *k*/(*mS*) (2)

where *k* is the kinetic constant from Formula (1), m is the mass of photocatalyst (0.1 g), and *S* is the specific surface area from Table 1. The apparent reaction rate constant (*K*) was calculated to be 0.0248, 0.0072, and 0.0027 min^−1^ for Cu_1.8_Se/Cu_3_Se_2_ composite, bare Cu_1.8_Se, and Cu_3_Se_2_, respectively. In other words, the photocatalytic activity per unit surface area of Cu_1.8_Se/Cu_3_Se_2_ is around 3.4 and 9.1 times higher than that of bare Cu_1.8_Se and Cu_3_Se_2_.

**Figure 6:**
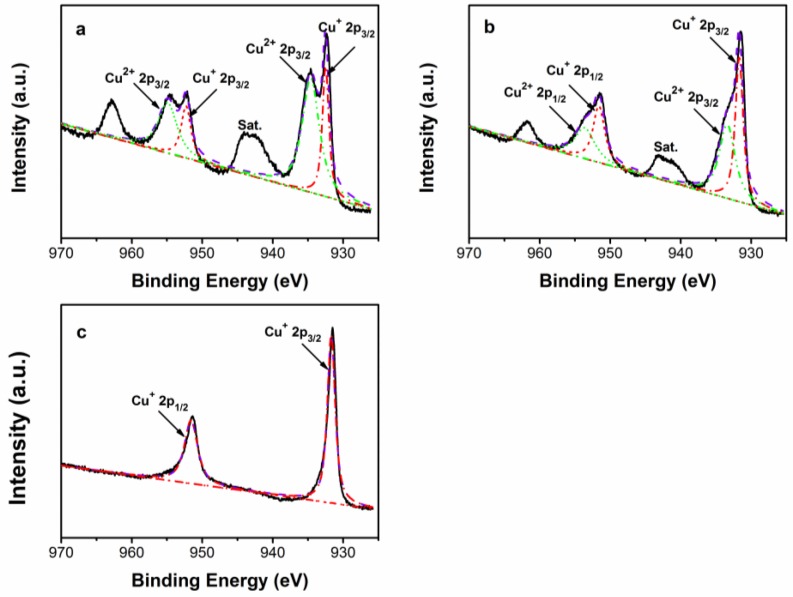
High-resolution X-ray photoelectron spectroscopy (XPS) scan of Cu 2p (**a**) Cu_3_Se_2_; (**b**) Cu_1.8_Se/Cu_3_Se_2_ composite; (**c**) Cu_1.8_Se.

On the other hand, X-ray photoelectron spectroscopy (XPS) analysis was carried out to investigate the chemical binding states of Cu_1.8_Se/Cu_3_Se_2_ composite, as shown in Figure 6a,b. The Cu 2p peaks of Cu_3_Se_2_ and Cu_1.8_Se/Cu_3_Se_2_ composite broaden and undergo splitting, while pronounced satellite peaks (marked as Sat.) form due to the existence of Cu^2+^ vacancy [40,41]. Two strong peaks are consistent with the literature data of Cu^+^ 2p_3/2_ and 2p_1/2_ [42,43,44]. On the sides of Cu^+^ 2p_3/2_ and 2p_1/2_ peaks, two low-intensity components appeared, which can be assigned to the Cu^2+^ oxidation state [45,46]. As illustrated in Figure 6c, the Cu 2p_3/2_ and Cu 2p_1/2_ peaks are symmetric, narrow, and devoid of satellite peaks, which is indicative of monovalent copper of Cu^+^ for bare Cu_1.8_Se. Therefore, the existence of Cu_3_Se_2_ leads to the presence of Cu^2+^ in the Cu_1.8_Se/Cu_3_Se_2_ composite.

In view of the mentioned XRD result (Figure 1b), Cu_1.8_Se is the main phase of Cu_1.8_Se/Cu_3_Se_2_ composite, resulting in the smaller relative concentration ratio of Cu^2+^ to Cu^+^ (1.04) than that (2.32) of bare Cu_3_Se_2_. Combined with the photodegradation results (Figure 3), it could be inferred that decreasing the concentration ratio of Cu^2+^ to Cu^+^ is conducive to enhancing photoactivity. It is worth noting that Cu_1.8_Se/Cu_3_Se_2_ composite has a larger concentration ratio of Cu^2+^ to Cu^+^ (1.04) than that (0) of bare Cu_1.8_Se, but the best photocatalytic activity—mainly due to the existence of phase junction between Cu_1.8_Se and Cu_3_Se_2_ mentioned in the HRTEM image (Figure 2d).

The relative band positions of the two semiconductors were determined to account for the enhanced photocatalytic activity of the Cu_1.8_Se/Cu_3_Se_2_ composite. The energy of conduction band (CB) bottoms (*E*_CB_) were calculated empirically according to formula [47]

*E*_CB_ = *X* − 0.5*E*_g_ + *E*_0_ (3)

where *E*_g_ is the band gap of the semiconductor, *E*_0_ is −4.5 eV for a normal hydrogen electrode, and X is the electronegativity of the semiconductor, expressed as the geometric mean of the absolute electronegativity of the constituent atoms [48]. The X values for Cu_1.8_Se and Cu_3_Se_2_ were calculated to be 4.94 and 5.0 eV, respectively, and the band gaps of Cu_1.8_Se and Cu_3_Se_2_ are 1.5 and 1.45 eV, respectively. On the basis of the equation above, the conduction band bottoms (*E*_CB_) of Cu_1.8_Se and Cu_3_Se_2_ were calculated to be −0.31 and −0.22 eV, respectively. Correspondingly, the valence band tops (*E*_VB_) of Cu_1.8_Se and Cu_3_Se_2_ are 1.19 and 1.23 eV, respectively. Thus, both the conduction band bottom (*E*_CB_) and the valence band top (*E*_VB_) of Cu_1.8_Se are higher than that of Cu_3_Se_2_. The calculated band positions of Cu_1.8_Se/Cu_3_Se_2_ composite was conducive to the separation and transportation of photogenerated carriers.

**Figure 7:**
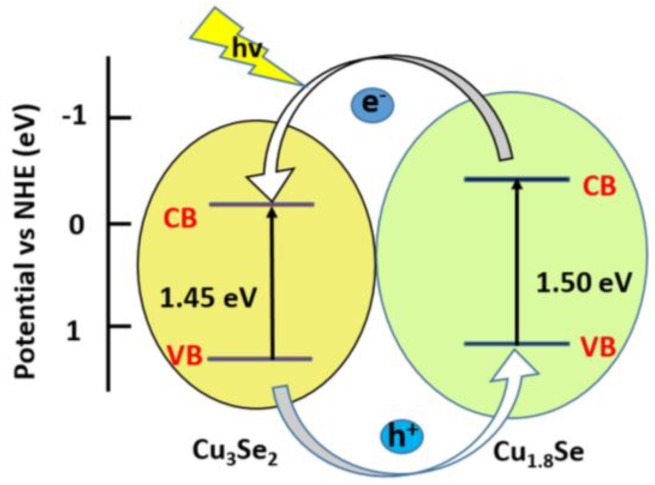
Diagram of energy band levels of Cu_1.8_Se/Cu_3_Se_2_ composites vs. NHE (normal hydrogen electrode) and the possible charge separation process.

As shown in Figure 7, Cu_1.8_Se and Cu_3_Se_2_ are easily excited by visible or NIR light, and photoinduced electrons and holes are generated. The CB edge potentials of the two phases enable photogenerated electrons to easily transfer from Cu_1.8_Se to Cu_3_Se_2_. Simultaneously, holes on the valence band of Cu_3_Se_2_ can be transferred to that of Cu_1.8_Se under the band energy potential difference. In such a way, long-lived reactive photogenerated carriers can be yielded, and thus enhanced charge separation efficiency through the phase junction can be achieved.

**Figure 8:**
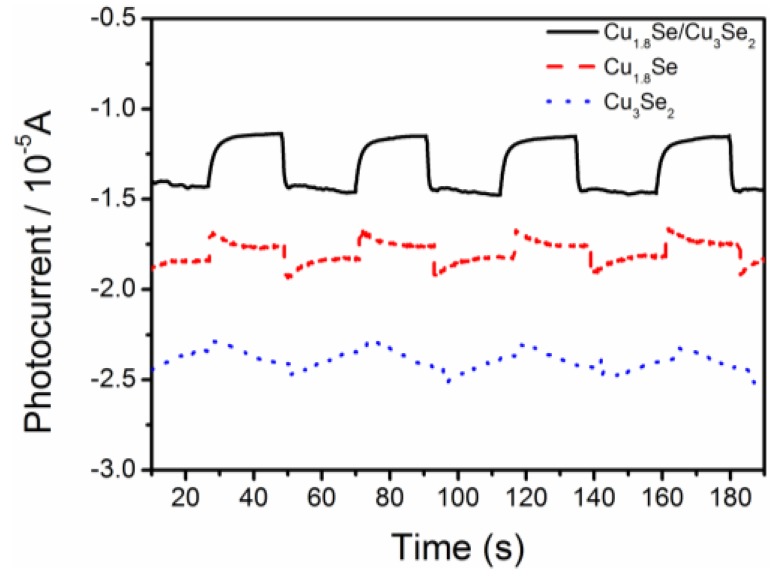
The photocurrent responses of copper selenides in 0.5 M Na_2_SO_4_ electrolyte under visible light.

To further confirm the effect of phase junction, the photoelectrochemical (PEC) behavior of Cu_1.8_Se/Cu_3_Se_2_ composite has been explored. As shown in Figure 8, the photocurrent responses were recorded under visible light irradiation. The electrodes (1 × 1 cm^2^) demonstrated photocurrent responses around 1 μA/cm^2^ and 2.5 μA/cm^2^ for Cu_1.8_Se and Cu_1.8_Se/Cu_3_Se_2_ composite, respectively, while the photocurrent responses of Cu_3_Se_2_ were not apparent. This result provides strong evidence that the formation of a phase junction between Cu_1.8_Se and Cu_3_Se_2_ would efficiently accelerate the separation efficiency of charge carriers. Therefore, the Cu_1.8_Se/Cu_3_Se_2_ composite presents highly enhanced performance as compared to bare Cu_1.8_Se and Cu_3_Se_2_.

## 3. Materials and Methods

### 3.1. Preparation

All reagents were of 99.9% purity and were used without further purification. A typical synthesis process of Cu_1.8_Se/Cu_3_Se_2_ composite was described as follows: 4 g NaOH, 0.76 g NaBH_4_, and 0.3 g elemental Se were added to 100 mL distilled water under constant stirring. The mixture reached about 80 °C in a few minutes to form alkaline selenium aqueous solution. Meanwhile, 10 mL Cu(NO_3_)_2_ aqueous solution (0.5 M) was prepared, and the mixture was combined with the alkaline selenium solution through rapid stirring. Finally, 0.1 g SDS was included as surfactant to control the morphology. After stirring for 8 h, the resulting products were separated by filtration, washed several times with distilled water and absolute alcohol, and then dried at 60 °C for 6 h. For comparison, bare Cu_3_Se_2_ and Cu_1.8_Se were also prepared by the hydrothermal method under the same conditions mentioned above by adding 0.4 g and 8 g NaOH, respectively.

### 3.2. Characterization

The phase and composition of the as-prepared samples was examined with an X-ray diffractometer (XRD, Bruker, D8 Advance, Beijing, China) using Cu Kα radiation (λ = 1.5418 Å). The morphology and microstructures were characterized by transmission electron microscope (TEM, JEOL JEM-2100, Beijing, China). X-ray photoelectron spectroscopy (XPS) measurements were conducted on a Thermo XPS ESCALAB 250Xi instrument (Thermo Scientific, Shanghai, China). UV-Vis-NIR diffuser reflectance (DRS) measurements were carried out on a UV/Vis/NIR spectrometer (PerkinElmer, Lambda 950, Shanghai, China). Specific surface area measurements were conducted by Autosorb-iQ2-Mp (Quantachrome, Shanghai, China).

### 3.3. Photocatalytic Test

The photocatalytic activities of as-prepared samples were investigated by photodegradation of MO under visible and NIR light. A 300 W xenon lamp (CEL-HXF300, Beijing, China) with cutoff filters (420 nm, 800 nm) was used as the light source. The specific process was as follows: 0.1 g photocatalyst was added into 100 mL MO (50 mg/L). Prior to irradiation, the slurry was continuously stirred in the dark for 1 h to ensure an adsorption–desorption equilibrium between photocatalyst and MO. Ice-water bath and magnetic stirring always existed to prevent thermal effect in the photocatalytic process. At a given time interval, 3 mL suspensions were centrifuged to remove the photocatalyst. The concentration of MO was analyzed by the UV-Vis spectrophotometer by recording the variations of the absorption band maximum (465 nm).

### 3.4. Photoelectrochemical Testing

Photocurrent under visible light was measured with a standard three electrode system on an electrochemical workstation (CHI 660, ChenHua, Shanghai, China). Ag/AgCl and Pt plate were used as reference electrode and counter electrode, respectively, in Na_2_SO_4_ solution (0.5 mol/L) as electrolyte. The working electrode was made by depositing photoctalyst on the FTO (fluorine-doped tin oxide) substrate (Beijing, China).

## 4. Conclusions

The Cu_1.8_Se/Cu_3_Se_2_ heterogeneous structure prepared by the simple precipitation method exhibits excellent photocatalytic activity in the degradation of MO under visible and NIR light irradiation, which is 3.4 and 9.1 times higher than that of bare Cu_1.8_Se and Cu_3_Se_2_. We also present that decreasing the concentration ratio of Cu^2+^ to Cu^+^ is advantageous for the enhancement of photoactivity. The mechanism of enhanced photocatalytic activity for the Cu_1.8_Se/Cu_3_Se_2_ composite was investigated based on the calculated energy band positions. The formation of a phase junction between the two semiconductors leads to the effective separation of photogenerated electron-hole pairs, which mainly account for the strongly enhanced photoactivity.

## Figures and Tables

**Table 1:** The BET (Brunauer-Emmett-Teller) specific surface area of different samples.

Samples	Cu_1.8_Se/Cu_3_Se_2_ Composite	Cu_1.8_Se	Cu_3_Se_2_
Size (nm)	66.3	48.6	57.8
Specific surface area (m^2^/g)	5.842	8.655	6.556
